# Exploring adolescents’ perspectives on and experiences with post-primary school-based suicide prevention: a meta-ethnography protocol

**DOI:** 10.1186/s13643-022-02166-1

**Published:** 2023-01-11

**Authors:** Eibhlin H. Walsh, Matthew P. Herring, Jennifer McMahon

**Affiliations:** 1 School, Child & Youth (SCY) Mental Health and Wellbeing Research Lab, Department of Psychology, University of Limerick, FG150, Foundation building, Limerick, Ireland; 2 National Institute of Studies in Education, University of Limerick, Limerick, Ireland; 3 Health Research Institute, University of Limerick, Limerick, Ireland; 4 Physical Activity for Health Cluster, Health Research Institute, University of Limerick, Limerick, Ireland; 5 Department of Physical Education and Sports Sciences, University of Limerick, Limerick, Ireland

**Keywords:** Post-primary school-based suicide prevention, Adolescents, Engagement, Involvement, Experiences, Perspectives, Evidence synthesis, Meta-ethnography protocol

## Abstract

**Background:**

Globally, suicide is the fourth leading cause of adolescent mortality. Although post-primary school-based suicide prevention (PSSP) interventions are an evidence-based strategy for targeting adolescent suicidal thoughts and behaviors (STBs), PSSP effectiveness does not easily translate to school settings. Adolescents’ perspectives on PSSP are particularly important for (1) intervention effectiveness and implementation in both research and practice, (2) addressing PSSP evidence-practice gaps, and (3) enhancing meaningful adolescent involvement in PSSP, yet there is a gap in understanding adolescents’ experiences of engaging with PSSP. As such, this protocol outlines a meta-ethnography which will explore and synthesize adolescents’ perspectives on engaging with PSSP interventions, as participants/end-users, intervention advisors, facilitators, and co-designers and co-researchers.

**Methods:**

The meta-ethnography protocol follows the Preferred Reporting Items for Systematic Review and Meta-Analysis Protocols (PRISMA-P) guidelines. The protocol was guided by the seven-stage process for meta-ethnography proposed by Noblit and Hare. Searches of PsycINFO, MEDLINE, Web of Science, CINAHL, ERIC, Scopus, and study reference lists will identify peer-reviewed studies. Gray literature will be identified by searches in ProQuest, British Library EThOS, and DART-Europe E-theses Portal. The main reviewer will initially assess the eligibility of studies based on title and abstract, with full texts reviewed by at least two reviewers. Findings of the included studies will be synthesized in line with Noblit and Hare’s stages and evaluated using the Critical Appraisal Skills Program (CASP) checklist.

**Discussion:**

To our knowledge, this is the first proposed meta-ethnography to explore and integrate the findings of qualitative studies exploring adolescents’ perspectives on engaging with PSSP interventions. Understanding adolescents’ experiences of engaging with PSSP will impact the field of PSSP in several ways by (1) enhancing research processes and intervention effectiveness and implementation, (2) informing decision-making and policymaking relevant to practice, (3) guiding meaningful adolescent involvement in PSSP, and (4) contributing to knowledge on the safety implications of engaging adolescents in PSSP. Finally, it is expected that the insights from this meta-ethnography will be widely applicable, given the growing demand for meaningful youth involvement in health-related fields.

**Systematic review registration:**

PROSPERO CRD42022319424.

## Background

Suicide is the fourth leading cause of mortality in 15–19-year-olds globally [1]. Concerns of increased youth suicide beyond COVID-19 [2–4] necessitates the implementation of effective adolescent suicide prevention strategies. In line with existing definitions of adolescence [5], we define adolescents as young people aged 11–19 years, which aligns with the typical age range of post-primary school students [6, 7]. Post-primary school-based suicide prevention (PSSP) is an evidence-based approach for targeting adolescent STBs [8, 9], which are prominent risk factors for death by suicide [10]. Despite the potential of PSSP to be a key adolescent suicide prevention approach, there are persistent challenges to translating the effectiveness of PSSP research to school settings, resulting in evidence-practice gaps [11, 12].

Adolescents’ experiences of engaging with PSSP are largely unexplored but are yet likely paramount to bridging the evidence-practice gap in PSSP. How acceptable and suitable health interventions are perceived by individuals engaged in interventions is critical to both intervention effectiveness [13] and implementation [14]. Engagement encompasses a variety of ways in which individuals are involved in interventions in research and practice, from participation as end-users to planning, design, analysis, translation, and dissemination [15, 16]. Adolescents not only engage with suicide prevention as research participants and intervention end-users, but also as intervention facilitators [17] and advisors [18, 19] and co-researchers [20].

A review of school-based mental health interventions supported intervention fidelity components linked to adolescents’ perspectives (i.e., receptiveness to interventions) as stronger predictors of postvention mental health outcomes, in comparison to intervention fidelity components linked to the intervention itself (i.e., intervention quality), indicating that adolescents’ perspectives on school-based mental health interventions are markedly important to intervention success [13]. The particular importance of understanding adolescents’ experiences of engaging with PSSP research is further exemplified by (1) the common delivery of PSSP in classrooms or to groups of adolescents who are developmentally more susceptible to peer influence [21, 22], (2) the links between adolescents’ difficulties in engaging with PSSP research as participants and participant drop out [23], (3) reports of a weak association between adolescents’ preintervention rates of STBs and perceiving a PSSP intervention as upsetting [24] and young people's perceptions of PSSP interventions as intrusive [25, 26], and (4) the sensitive nature of suicide as a mental health topic [27].

Furthermore, understanding adolescents’ perspectives on PSSP thus far is a fundamental step in progressing meaningful adolescent engagement in PSSP research, which can be understood as adolescents’ active and decisive involvement throughout the research process [28]. One reason that patient and public involvement in research is gaining traction in recent times [29] is that interventions are more acceptable and suitable when target populations are meaningfully engaged in the research process [30]. However, meaningful adolescent engagement in PSSP research disproportionately lacks; for the most part, adolescents did not aid the design of or input on PSSP interventions in a recent review of studies investigating the impact of PSSP interventions on STBs [31] and partnerships between suicide prevention researchers and young people are sparse [32]. Adultism has positioned young people passively in research leading to lack of agency and space for young people to contribute to research meaningfully [33]. Young people are well-positioned to advise on and partake in decision-making and design processes in suicide prevention [34, 35] and have expressed the importance of their voice in school mental health [36].

Moreover, knowledge of adolescents’ perspectives on PSSP and enhancing meaningful adolescent involvement in PSSP research also responds to moral and socio-political obligations; the United Nation’s Convention on the Rights of the Child mandates that adolescents’ views are forefront to matters concerning them [37], and the Lancet Commission on Adolescent Health and Wellbeing advocates for adolescent voice in interventions concerning their well-being [38].

There is a critical need to address the gap in understanding adolescents’ perspectives on engaging with PSSP. There is no known synthesis of the qualitative research exploring and collating the varied experiences of young people engaging with PSSP, despite the existence of qualitative research exploring adolescents’ perspectives on engaging with PSSP. Meta-ethnography is well-positioned to draw qualitative findings together, generate over-arching understandings of collective experiences, and develop insights to inform decision-making [39].

### Aim

The aim of the current protocol is to outline a meta-ethnography review, which will explore adolescents’ perspectives on and experiences of engaging with PSSP interventions, as participants/end-users, intervention advisors, facilitators, and co-designers and co-researchers. The research questions guiding the meta-ethnography were developed based on the Population, Exposure and Outcome (PEO) framework [40] and are as follows:

1. What are the perspectives of adolescents aged 11–19 years on engaging with PSSP interventions, as participants/end-users, intervention advisors, facilitators, and co-designers and co-researchers?
2. What are the experiences of adolescents aged 11–19 years in engaging with PSSP interventions, as participants/end-users, intervention advisors, facilitators, and co-designers and co-researchers?

## Methods/design

The Preferred Reporting Items for Systematic Review and Meta-Analysis Protocols (PRISMA-P) guidelines [41] informed the preparation and the reporting of the present meta-ethnography protocol. This protocol is registered with PROSPERO (CRD42022319424).

Qualitative studies will be identified in the review and subject to a meta-ethnographic approach. Meta-ethnography will be used to synthesise and evaluate the outcomes of the included studies, in line with Noblit and Hare’s (1988) seven stage process for conducting meta-ethnography, which includes the following stages (1) getting started, (2) describing what is relevant to the initial interest, (3) reading included studies, (4) determining how the studies are related, (5) translating the studies into one another, (6) synthesising translations, and (7) expressing the synthesis. The meta-ethnography will also be guided by Campbell and colleagues [42], Lee and colleagues [43], and France and colleagues and will be presented in line with the eMERGe reporting guidance and the PRISMA statement guidelines [44, 45].

### Study selection

Consistent with the PEO framework studies were eligible for inclusion if they sampled adolescents aged 11–19-year-olds who had engaged with PSSP interventions as participants/end-users, intervention advisors, facilitators, and co-designers and co-researchers (“Population”); included interventions conducted in post-primary school settings targeting suicide-related outcomes (i.e., STBs, help-seeking behaviors) as both primary intervention outcomes and with other health and well-being outcomes (“Exposure”); and reported adolescents’ experiences with and perspectives on engaging with PSSP (“Outcome”).

Inclusion criteria include (1) English language studies, (2) peer-reviewed journal articles and gray literature, and (3) qualitative and mixed-method studies reporting qualitative data collection methods and qualitative analysis. Exclusion criteria include (1) studies which do not include a method of qualitative data collection or analysis, (2) review and synthesis studies, and (3) studies which sample participants engaging with PSSP located in third-level education or university settings. Third-level education refers to education after second-level schooling, which results in a level 4 + qualification (i.e., university degree or degree apprenticeship [46]).

Studies available from database inception will be identified by searches in PsycINFO, MEDLINE, Web of Science, CINAHL, ERIC, and Scopus. Gray literature will be identified by searches in ProQuest, British Library EThOS, and DART-Europe E-theses Portal. Where appropriate, MeSH, subject, and wildcard terms and truncation will be applied to database search strings. Database searches will be supplemented by searches in Google Scholar and reference lists of screened studies. Table 1 outlines the search terms which will be used for the literature searches. If full texts of studies are unavailable the corresponding author will be contacted, with a period of 21 days the maximum waiting time for studies. Retrieved studies from database searching will be exported to RAYYAN QCRI [47].

**Table 1:** Search terms for database searches

PEO framework components	Search terms
Population	adolescen* or teen* or young and adult* or youth or student or child*
Exposure	education* or school OR high? school or secondary school OR post? primary or school? based or middle? school and prevention or intervention or program* and suicid*
Outcomes	perspective* or perception* or attitude* or perceive* or understanding or experience* or view* or opinion

Search terms will be used for Scopus and adapted for other databases

Initially, the main reviewer will assess the eligibility of studies based on title and abstract. Any uncertainty of eligibility will be discussed by the reviewers until consensus is met. Then, the main reviewer will assess the eligibility of all full-texts, with full-texts reviewed by at least two reviewers. Any discrepancies in full-text assessment will be discussed with the aim of reaching consensus. If necessary, where consensus cannot be met between the main reviewer and the other reviewer, a third reviewer will be consulted. Inter-rater reliability of full text assessment will be quantified using Cohen’s Kappa Measure of Agreement Coefficient [48]. All excluded articles (including duplicates) will be recorded. This selection process will be detailed in the PRISMA flow diagram for systematic reviews [45].

### Quality appraisal

Study appraisal will be undertaken using CASP checklists [49], which will be completed by two reviewers, to both assess quality of the included studies and enhance in-depth understanding of studies. An additional question will be added to the CASP assessment; “Are the interventions of interest clearly described?”, similar to the procedure of a meta-ethnography synthesis of young people’s experiences with mental health interventions [50]. The consideration to data saturation as part of the CASP checklist criteria will only be given to studies employing qualitative methodologies which recognise data saturation as an appropriate conceptual tool [51]. Any disagreement in CASP assessments between reviewers will be discussed until consensus is reached. If necessary, where consensus cannot be met between the two reviewers, a third reviewer will be consulted.

### Stage (1): getting started

The topic of interest was identified and discussed by the co-authors and explored based on the literature presented in the “Background” section of this protocol. Meta-ethnography was identified as an appropriate methodology to synthesize qualitative research exploring experiences and perspectives of adolescents engaging with PSSP [39]. Furthermore, meta-ethnography enables the generation of novel interpretations which both explain and go beyond the findings of individual studies included in the review [42, 52].

### Stage (2): describing what is relevant to the initial interest

The end-goal of this stage is to develop an exhaustive list of studies to be included in the meta-ethnography [39], which will be achieved by study selection procedures.

### Stage (3): reading included studies

Reading the included studies will be undertaken to achieve several goals, including familiarization, extraction, appraisal, and comparison [43]. The main reviewer will also partake in active reading by annotating and coding data, to facilitate in depth appraisal of the studies, as recommended by Lee and colleagues [43]. Studies which lack conceptual depth will not be synthesized, as recommended by France and colleagues [44].The main reviewer will extract participant and study author data relating to adolescents’ experiences with and perspectives on PSSP. Participant data includes participants’ own understanding of beliefs and experiences (i.e., first-order constructs) and study author data includes study authors’ interpretations of participant data (i.e., metaphors, themes, categories, concepts, ideas, metaphors etc.) (i.e., second-order constructs) [39, 42, 43]. The following data from the included studies will be tabulated and described in the main text of the meta-ethnography: (1) Study and intervention characteristics (study aims and intervention types), (2) participant demographics and characteristics, (3) school demographics and characteristics, and (4) data collection and analysis details (i.e., data collection method and analytical approach). NVivo 12 software will record data.

### Stage (4): determining how the studies are related

The main reviewer will lead data analysis, and at least one other reviewer will provide critique and guidance on analysis, in line with Lee and colleagues’ recommendations of enriching meta-ethnographic interpretation through collaboration. Stage 4 will involve three key steps [53]: (1) listing first- and second-order constructs and documenting how constructs relate to each other within study accounts, (2) comparison of constructs and study characteristics across studies, and (3) determining how key constructs relate to one another across studies. Firstly, first- and second-order constructs will be coded using an a priori coding frame based on the research questions, similar to previous meta-ethnography procedures [55]. Given that study authors report participants’ quotations to support their interpretations of the data, first-order constructs will be coded (and subsequently analyzed and synthesized) alongside corresponding second-order constructs [53, 54]. Commonality and reoccurrence between constructs within studies will be recorded*.* Secondly, coded constructs (and within-study relationships) will be compared across studies. Key themes representing common and reoccurring constructs sharing underlying central concepts will be developed. Study characteristics including research design, intervention characteristics, and participant characteristics will be subject to cross study comparison. Thirdly, how studies relate to one another (i.e., reciprocally and/or refutational) will be determined based on the relationships between studies and key themes. Studies will be grouped based on the presence or absence of key themes. The relationships between key themes will also be considered in relation to the research questions and study characteristics [42, 43, 53].

### Stage (5): translating the studies into one another

Stage 5 will involve the translation of constructs from one study into another to arrive at constructs which embody multiple constructs [42, 53]. The identification of similarities and disagreements between studies will guide the subsequent synthesis approaches (i.e., reciprocal translations and refutational synthesis) [39, 42, 53]. Studies will be translated into each other by conducting a constant comparison of the grouped studies, which involves conducting between-study comparisons, while maintaining within-study comparisons; for example, key constructs and themes in study one will be compared with key constructs and themes in study two, and key constructs and themes in study one and study two will be compared with constructs and themes in study three [43, 53, 54].

### Stage (6): synthesizing translations

The type of synthesis approach undertaken will determine if constructs and their respective translations encompass those in other studies, leading to analysis of competing translations and translation into each other. Stage 6 will result in key meta themes and a line-of argument synthesis will be created from the resulting third-order constructs. Line-of-argument synthesis may lead to a new model or theory beyond the individual interpretations of the included studies, about the experiences of young people engaging with PSSP [39, 43, 53].

### Stage (7): expressing the synthesis

The synthesis will follow the eMERGe reporting guidance and will be submitted for peer review publication. It is expected that key findings from the meta-ethnography will be presented to academic and lay audiences.

## Discussion

To our knowledge, this is the first proposed meta-ethnography which will explore and synthesize qualitative studies documenting adolescents’ perspectives on engaging with PSSP, as participants/end-users, intervention advisors, facilitators, and co-designers and co-researchers. Insights from this meta-ethnography will address a key gap in the field of PSSP by enhancing understanding of the experiences of adolescents’ engaging with PSSP, such as how acceptable and suitable (or not) PSSP may be perceived by adolescents and the potential barriers to and facilitators of engaging with PSSP. The generation of a “line-of-argument” will result in a novel and overarching understanding of adolescents’ experiences with PSSP, which may reveal interpretations which were not apparent in the individual studies [39]. These insights have potential to have considerable impact in guiding decision-making and policymaking in the field of PSSP [42], which could be formative in addressing the longstanding PSSP evidence-practice gap.

Given that qualitative analysis of experiences is critical for informing intervention research and highlighting important information relating to intervention context and implementation [56, 57], the application of insights generated from this meta-ethnography could contribute to enhancing PSSP research outcomes, intervention effectiveness, and implementation in practice. Furthermore, it is expected that insights from this meta-ethnography will inform guidance on *how* adolescents should be involved in PSSP, as participants/end-users, intervention advisors, facilitators, and co-designers and co-researchers, which is necessary to alter the trajectory of adolescents’ lack of meaningful involvement in PSSP research. No known comprehensive guidance of this kind exists.

To enhance meaningful youth involvement in youth mental health research in general, research must move away from a focus of young people engaging in research as passive subjects, towards a view of young people as central to research [28]. Collated insights on how adolescents perceive their involvement in PSSP research, generated through meta-ethnography, will be pivotal for re-focusing the PSSP research field towards the experiences of adolescents involved in PSSP. Given that meta-ethnography allows for the identification of insights beyond individual studies, potential absences, and misinterpretations of knowledge in the literature may be highlighted [39, 42], which is particularly important to counter adultism in research and the fact that adolescents’ perspectives are typically presented through adult lenses in research [58].

Although evidence does not indicate harmful effects after exposure to suicide research in assessment [59], screening [60, 61], content [62]; and participatory-based research [20, 63, 64], risks of harm of researching suicide remains a steadfast barrier for progressing research on suicide prevention [27, 60], particularly with young people [20, 65]. Insights from this meta-ethnography are essential to understanding the safety implication of engaging adolescents in PSSP.

There is increasing momentum for understanding the involvement of young people involved in research in wider-health-related fields [66]. As such, it is expected that the insights from this meta-ethnography will transcend the field of PSSP, given the international calls for enhancing meaningful youth voice and involvement in youth in suicide prevention [34], school mental health practice [67], and wider-health related research [38].

With respect to operational issues, initially adolescents’ experiences with and perspectives on PSSP will be synthesized with respect to whether adolescents engaged with PSSP as participants/end-users, intervention advisors, facilitators, and co-designers and co-researchers. Data will be fully integrated, similar to the procedure of Evans and Hurrell [56], provided that *how* adolescents engaged with PSSP is subordinate to the similarities and differences across the studies. Finally, amendments to the protocol will be described in the completed review publication.

## Data Availability

Not applicable.

## Abbreviations

PSSP: Post-primary school-based suicide prevention
STBs: Suicidal thoughts and behaviors
PRISMA-P: Preferred Reporting Items for Systematic Review and Meta-Analysis Protocols
CASP: Critical Appraisal Skills Program
